# Characterization of Plant Volatiles Reveals Distinct Metabolic Profiles and Pathways among 12 Brassicaceae Vegetables

**DOI:** 10.3390/metabo8040094

**Published:** 2018-12-14

**Authors:** Yu Liu, Hui Zhang, Shivshankar Umashankar, Xu Liang, Hui Wen Lee, Sanjay Swarup, Choon Nam Ong

**Affiliations:** 1 NUS Environment Research Institute, National University of Singapore, Singapore 117411, Singapore; eriliu@nus.edu.sg (Y.L.); erizh@nus.edu.sg (H.Z.); shivshankar.nus@gmail.com (S.U.); eriliaxu@nus.edu.sg (X.L.); erilhw@nus.edu.sg (H.W.L.); sanjay@nus.edu.sg (S.S.); 2 Saw Swee Hock School of Public Health, National University of Singapore, Singapore 117549, Singapore

**Keywords:** VOCs profiling, cruciferous vegetables, HS-SPME, GC-HRMS, metabolic pathway, chemotaxonomy

## Abstract

Plants emit characteristic organic volatile compounds (VOCs) with diverse biological/ecological functions. However, the links between plant species/varieties and their phytochemical emission profiles remain elusive. Here, we developed a direct headspace solid-phase microextraction (HS-SPME) technique and combined with non-targeted gas chromatography‒high-resolution mass spectrometry (GC-HRMS) platform to investigate the VOCs profiles of 12 common Brassicaceae vegetables (watercress, rocket, Brussels sprouts, broccoli, kai lan, choy sum, pak choi, cabbage, Chinese cabbage, cauliflower, radish and cherry radish). The direct HS-SPME sampling approach enabled reproducible capture of the rapid-emitting VOCs upon plant tissue disruption. The results revealed extensive variation in VOCs profiles among the 12 Brassicaceae vegetables. Furthermore, principal component analysis (PCA) showed that the VOC profiles could clearly distinguish the 12 Brassicaceae vegetables, and that these profiles well reflected the classical morphological classification. After multivariate statistical analysis, 44 VOCs with significant differences among the Brassicaceae vegetables were identified. Pathway analysis showed that three secondary metabolism pathways, including the fatty acid pathway, methylerythritol phosphate (MEP) pathway and glucosinolate (GLS) pathway, behave distinctively in these vegetables. These three pathways are responsible for the generation and emission of green leaf volatiles (GLVs), terpenes and isothiocyanates (ITCs), respectively. Correlation analysis further showed that volatile metabolites formed via the common pathway had significantly positive correlations, whereas metabolites from different pathways had either non-significant or significantly negative correlations. Genetic influences on these metabolites across various vegetable types were also evaluated. These findings extend our phytochemical knowledge of the 12 edible Brassicaceae vegetables and provide useful information on their secondary metabolism.

## 1. Introduction

Plants produce an enormous spectrum of volatile organic compounds (VOCs) as secondary metabolites with diverse biological properties and functions. In general, plant VOCs are low molecular weight metabolites with low boiling point and high vapour pressure at ambient temperature. Plant VOCs are usually categorized into terpenoids, fatty acid derivatives, amino acid derivatives and benzenoid compounds [1]. These volatiles can be emitted from flowers, leaves, fruits, and roots into the atmosphere or soil, allowing the plant to interact with its surroundings [2]. From a physiological standpoint, plant VOCs play important roles in plant‒plant or within-plant signalling [3,4], as well as providing protection by deterring herbivores or pathogens [5,6], attracting natural enemies of herbivores [7], and enhancing resistance to plant damage or against abiotic stresses [8]. In addition, plant VOCs are major determinants of food flavour and aroma, which are of great agronomic importance for food quality [9,10]. Recently, the findings of the beneficial effects of plant VOCs on human health also shed light on drug discovery [11]. Thus, plant VOCs, as a significant part of plant metabolome, are of particular interest to researchers in ecology, agriculture, food quality and safety, and functional food usage. 

Brassicaceae vegetables, also known as cruciferous vegetables, have attracted increasing research interest because they are among the most commonly consumed vegetables globally, and have been demonstrated to have substantial human health benefits [12]. Recent studies on VOCs emitted from Brassicaceae vegetables have been dedicated to characterizing the vegetables, identifying the aroma components, and evaluating the quality of vegetables, such as VOCs in watercress [13], radish [14], Korean cabbage [15], Brussels sprouts and cauliflower during blanching [16], Brussels sprouts damaged by caterpillars [17], broccoli under ambient and elevated atmospheric CO_2_ concentrations [18] and rocket during the post-harvest storage [19]. However, most of these studies focused mainly on the fingerprint of VOCs in a single species, so there is still a lack of comprehensive comparison of VOCs profiles in commonly consumed Brassicaceae vegetables. As volatile metabolites in vegetables, VOCs are typical end products in the related metabolic pathways. For example, studies revealed that the glucosinolates (GLSs) in Brassicaceae vegetables could be enzymatically broken down to form isothiocyanates (ITCs), which are typical vegetable VOCs [7,20]. The VOCs profiles may reflect differences in metabolic pathways among various vegetables. In Brassicaceae vegetables, the correlation of VOCs and the possible metabolic pathways for the formation and emission of VOCs are yet to be systematically investigated. 

On the other hand, there has been significant progress in the methodologies and analytical instruments for precise and comprehensive investigation of plant VOCs. In the literature, various extraction methods have been developed for analysis of VOCs in plant, including liquid-liquid extraction [21], steam distillation or simultaneous distillation extraction [22], purge and trap, and headspace solid phase micro-extraction (HS-SPME) [23]. The HS-SPME technique employs a small fibre coated with an adsorbing phase, and has found widespread applications in qualitative and quantitative analyses of VOCs in various food, environmental and biological samples [23,24,25]. Comparing with other extraction methods, HS-SPME is less destructive to the samples and can capture the VOCs in a short time. These advantages make HS-SPME a promising technique for rapidly extracting VOCs that are rapidly released from fresh vegetables upon cell rupture. It is known that plant VOCs are chemically instable and the profiles may change rapidly after leaf and stem damage [26]. To elucidate the related metabolic pathways in vegetables, it is crucial to obtain VOCs in a fast and gentle way. For this purpose, direct HS-SPME may be a good choice and deserves further investigation. In particular, the combination of HS-SPME with gas chromatography-high resolution mass spectrometry (HRMS) can provide reliable and high-quality VOCs profiles in a simple and less tedious way. 

The objectives of this study are (i) to develop an effective sampling method for determining the VOCs released from 12 different Brassicaceae vegetables, using HS-SPME combined with GC-HRMS; (ii) to find out whether the VOCs profiles can be used to distinguish various Brassicaceae vegetables; and (iii) to elucidate the possible metabolic pathways, and their relationships associated with the metabolism of VOCs. 

## 2. Results

### 2.1. Optimization of VOCs Extraction 

The extraction temperature, extraction time and addition of water during blending were optimized for extraction of vegetable VOCs through HS-SPME by using broccoli as a model vegetable. Extraction efficiencies of VOCs at different temperatures (20 °C and 37 °C) were evaluated (Figure S1a). Data suggested that more peaks and higher peak abundances were observed for extraction of the broccoli sample at 37 °C as compared to extraction at 20 °C. Moreover, VOCs profiles at 37 °C showed higher reproducibility in total ion chromatograms (TICs). Therefore, in order to obtain more comprehensive volatile information all subsequent incubations and extractions were conducted at 37 °C.

Different extraction times (30, 60, 90 and 180 min) were also examined as the releasing rate of diverse VOCs may vary significantly. In this study, we were more interested in the rapid-emitting VOCs, thus we implemented the SPME extraction immediately after the vegetable tissues were disrupted in an enclosed sampling system. Although there were more peaks detected with increasing extraction time (Figure S1b), most of these VOCs were eluted after 11 min. These late-eluted peaks were VOCs with relatively high molecular weight and high boiling point. Nevertheless, it was noted that the profiles before 11 min were constituents with lower molecular weight and boiling point not affected significantly by increasing sampling time. Moreover, a long sampling time may sacrifice the reproducibility and performance of the SPME fibre. In view of these reasons, 30 min was thus selected as the extraction time for subsequent studies.

Adding a small amount of water into the vegetable samples during blending might help homogenize the tissue to enhance extraction, thus both conditions, i.e., with or without water added, were evaluated during preliminary sample treatment investigations. It was noted that addition of water would help homogenize the vegetable to some extent. However, the addition of water led to less peaks extracted from the vegetable at 37 °C for 30 min (Figure S1c), which was probably due to the dissolution of VOCs in water. 

Thus, throughout this VOCs profiling investigation, all the vegetables were extracted at 37 °C for 30 min without addition of water during blending. A typical TIC of VOCs extracted from crushed broccoli tissues under optimal extraction conditions is displayed in Figure S1d, which demonstrates a comprehensive capture of various VOCs emitted from the plant tissues. 

### 2.2. Classification of VOCs in 12 Brassicaceae Vegetables

**Figure 1:**
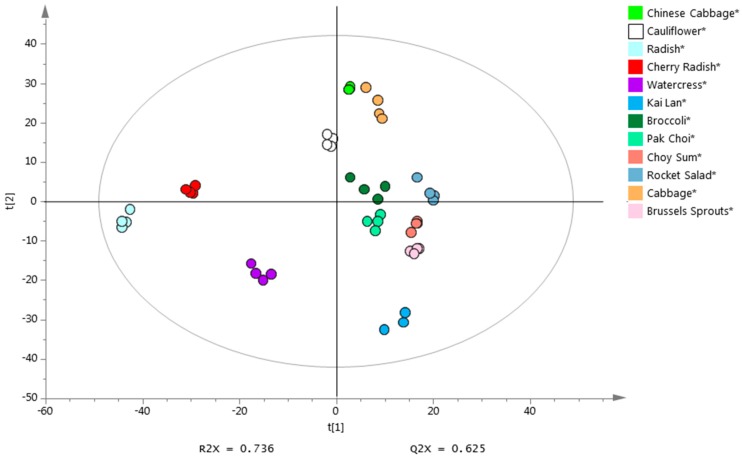
PCA analysis based on VOCs emitted from 12 Brassicaceae vegetables.

PCA was performed to assess whether the emitted VOCs can be used to distinguish the twelve vegetables in the Brassicaceae family. After removing features with low DFs and high RSDs, as well as common features detected in blanks with mean abundance higher than half the mean abundance in samples, there were 1490 features subjected to PCA. As shown in Figure 1, 12 distinct clusters each consisting of four vegetable replicates were well separated in the PCA scores plot (PC1 × PC2). The results showed that these 12 types of vegetables could be grouped according to their morphological taxonomic subfamilies (Figure 1), i.e., *Brassica* (*Brassica oleracea gemmifera*, *Brassica olearacea italic*, *Brassica olearacea alboglabra*, *Brassica rapa parachinensis*, *Brassica rapa chinensis*, *Brassica oleracea capitata*, *Brassica rapa pekinensis* and *Brassica oleracea botrytis*), *Raphanus* (*Raphanus sativus* and *Raphanus raphanistrum sativus*), *Nasturitum* (*Nasturtium officinale*) and *Eruca* (*Eruca sativa*). This finding reveals a link between morphological taxonomic classification and VOC-based chemotaxonomic classification, suggesting that the VOC profiles may have the potential to be used as phenotypic makers to characterize species in Brassicaceae family. 

### 2.3. Differential VOCs Compositions in 12 Brassicaceae Vegetables

The VOCs contributing to discriminate between the 12 Brassicaceae vegetables were screened using multivariate statistical analysis and identified by comparison to NIST library (11.0). Information of the identified VOCs, including names, CAS no., retention time, retention indices, VIP and *p* values is included in Table S1. In total, 44 VOCs were identified, including two alkanes, five alcohols, four aldehydes, seven esters, seven terpenes, 10 isothiocyanates, three nitriles, and six sulphur compounds. Five of the identified VOCs were further confirmed by authentic standards (allyl isothiocyanat, 3-butenyl isothiocyanate, PEITC, 3-(methylthio) propyl isothiocyanate and dimethyl disulphide). 

**Figure 2:**
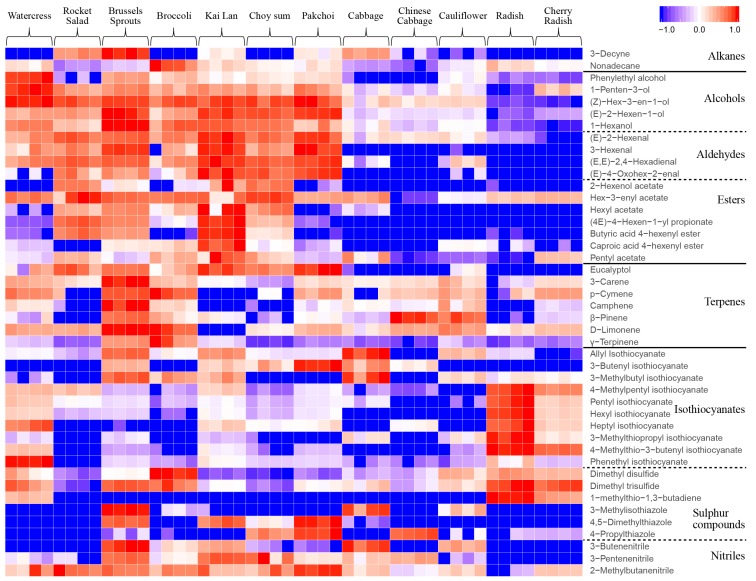
Heatmap of VOCs emitted from 12 Brassicaceae vegetables. The levels of each VOC in the 12 vegetables were normalized in the range of −1 to 1. Blue (−1) and red (1) represent the lowest and highest levels, respectively.

A heatmap (Figure 2) based on the abundance of VOCs was derived to compare their relative levels in the 12 vegetables. Similar VOCs profiles were found in watercress (*Nasturtium officinale*), rocket (*Eruca sativa*), Brussels sprouts (*Brassica oleracea var. gemmifera*), broccoli (*Brassica olearacea var. italic*), kai lan (*Brassica olearacea var. alboglabra*), choy sum (*Brassica rapa var. parachinensis*) and pak choi (*Brassica rapa subsp. Chinensis*), which had relatively higher levels for most of the VOCs detected as compared to other Brassicaceae vegetables studied. Cabbage (*Brassica oleracea var. capitata*), Chinese cabbage (*Brassica rapa subsp. Pekinensis)* and cauliflower (*Brassica oleracea var. botrytis*) showed similar VOCs profiles, with relatively low levels. By contrast, radish (*Raphanus sativus*) and cherry radish (*Raphanus raphanistrum subsp. Sativus*) both had low levels of VOCs, except for several isothiocyanates and sulphur compounds, which were at relatively higher levels as compared to other vegetables. 

In addition, the heatmap also demonstrates that among the 12 Brassicaceae vegetables, the identified VOCs in different chemical groups (e.g., alcohols, aldehydes and esters; terpenes; isothiocyanates; nitriles; sulphur compounds) showed distinct profiles (Figure 2). The detection of various VOCs from different chemical groups in the 12 vegetables are described below. 

*Alcohols*, *aldehydes and esters*. As shown in Figure 2, these VOCs were present in relatively high levels in watercress, rocket, Brussels sprouts, broccoli, kai lan, choy sum, and pak choi, which are usually referred to as green leafy vegetables grown above ground. In contrast, these VOCs were in very low levels in radish and cherry radish, which are root-type vegetables that grown below ground. The levels of alcohols, aldehydes and esters in cabbage, Chinese cabbage and cauliflower were also low, but were relatively higher than root-type vegetables. The C6 aldehydes, alcohols, and their esters are specifically referred to as green leaf volatiles (GLVs) [1]. In this study, the detected GLVs included 1-hexanol, 3-(Z)-hexenol, 2-(E)-Hexenol, 2-(E)-hexenal, 3-hexenal and 2,4-(E,E)-hexadienal, 2-hexenol acetate, (3Z)-3-hexenyl acetate, hexyl acetate, (4E)-4-hexenyl propionate, (4E)-4-hexenyl butyrate, and (4E)-4-hexenyl hexanoate. 

*Terpenes.* Six monoterpenes (3-carene, p-cymene, camphene, β-pinene, D-limonene and γ-terpinene) were identified with significant variations among the 12 Brassicaceae vegetables (Figure 2). These monoterpenes were found to be most abundant in Brussels sprouts, followed by broccoli, whereas the lowest levels were present in radish and cherry radish. 

*Isothiocyanates*. Ten ITCs were identified in the 12 Brassicaceae vegetables (Figure 2). The highest levels of several ITCs, including 4-methylpentyl ITC, pentyl ITC, hexyl ITC, heptyl ITC, and 3-methylthiopropyl ITC were found in radish. Allyl ITC, 3-butenyl ITC, and 3-methylbutyl ITC were high in cabbage, and PEITC was the highest in watercress. Relatively lower levels of ITCs were found in rocket, broccoli, cabbage, Chinese cabbage and cauliflower. 

*Nitriles*. As for nitriles (Figure 2), 3-butenenitrile, 3-pentenenitrile and 2-methyl butane nitrile were diversely distributed in the 12 Brassicaceae vegetables and higher levels of these nitriles were found in Brussels sprouts compared to other vegetables. Although the root vegetables, i.e., radish and cherry radish, had very high levels of ITCs, they had the lowest levels of nitriles. 

*Sulphur compounds*. Notably, varied emissions of dimethyl disulphide, dimethyl trisulfide, 1-(methylsulfanyl)-1, 3-butadiene, 3-methylisothiazole, 4,5-dimethylthiazole, and 4-propylthiazole among the 12 Brassicaceae vegetables were observed (Figure 2). Relatively high levels of sulphur compounds were detected in Brussels sprouts and low levels were mainly observed in watercress and rocket. It was noted that radishes and cherry radishes had high levels of dimethyl disulphide, dimethyl trisulfide and (1E)-1-(methylsulfanyl)-1,3-butadiene, but low levels of the other three sulphur compounds (3-methylisothiazole, 4,5-dimethylthiazole, 4-propylthiazole). 

### 2.4. Metabolic Pathways of VOCs

**Figure 3:**
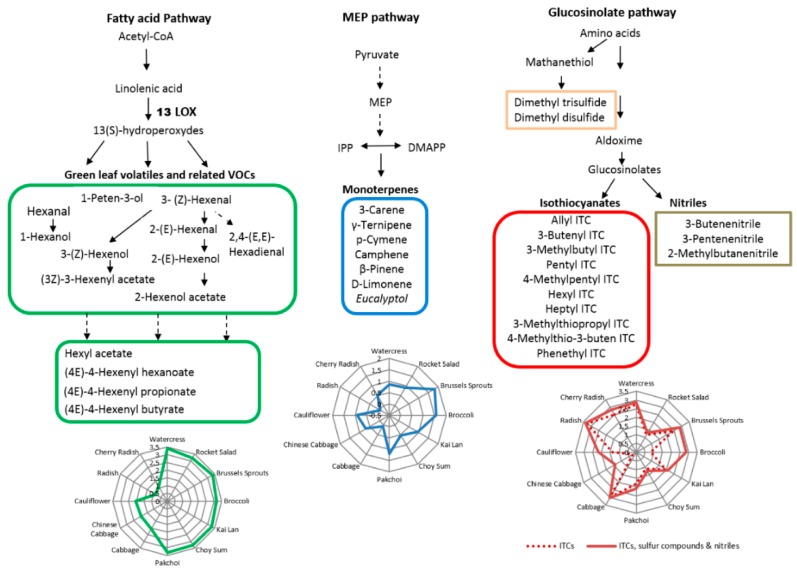
Main metabolic pathways of VOCs and their distribution in 12 Brassicaceae vegetables.

Based on the literature, there are several biosynthetic pathways leading to the diverse range of plant VOCs. The three main metabolic pathways involved in the formation of the identified VOCs in this study are summarized in Figure 3. The metabolic pathways included fatty acid pathway (for secondary volatile metabolites, e.g., GLVs), MEP pathway (for terpenes), and glucosinolate pathway (for ITCs/nitriles). The GLVs are known to be produced from C18 unsaturated fatty acids including linoleic acid or linolenic acid through the lipoxygenase (LOX) pathway [1]. Our results showed that green leafy vegetables (e.g., watercress, rocket, Brussels sprouts, broccoli, kai lan, choy sum, and pak choi) generated more GLVs, indicating that the LOX pathway may behave differently between these green leafy vegetables and others. Monoterpenes are synthesized mainly by the MEP pathway in plastids [1], and our results showed that Brussels sprouts and broccoli may generate more monoterpenes than other vegetables through this pathway. ITCs and nitriles are hydrolysis products of GLSs in plants, which are metabolized from their corresponding precursor amino acids such as alanine, leucine and methionine [27]. Methionine is also the precursor of numerous sulphide compounds including dimethyl trisulfide and dimethyl disulphide [28]. In our study, radish, cherry radish, cabbage, watercress and Brussels sprouts emitted higher levels of volatile ITCs, and broccoli, Chinese cabbage and cauliflower emitted higher levels of nitriles and sulphur compounds.

### 2.5. VOC Differences Arise from Variation in Genomic Potential of Brassicaceae

In order to understand the differences in VOC compositions, specifically in relation to fatty acid pathway (for secondary volatile metabolites, e.g., GLVs), MEP pathway (for terpenes), and glucosinolate pathway (for ITCs/nitriles), we performed comparative pathway analysis between *Brassica oleracea capitate*, *Brassica oleracea oleracea*, *Brassica rapa pekinensis* (using PlantCyc databases https://www.plantcyc.org/) [29] and *Raphanus sativum* (using RadishBase) [30]. There are multiple sub-pathways that differ between these four species (Table S2), with the pathways of interested being colour-coded. 

**Figure 4:**
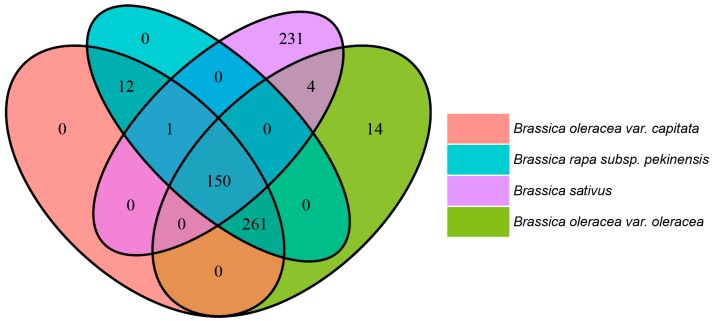
Metabolic pathway differences in *Brassica oleracea var. capitata*, *Brassica oleracea var. oleracea*, *Raphanus sativus* and *Brassica rapa subsp. Pekinensis* based on comparative genomics. The Venn diagram shows the similarity and differences between metabolic pathways among the four plant species: *Brassica oleracea var. capitata* (in orange), *Brassica olearacea var. oleracea* (in green), *Brassica rapa subsp. Pekinensis* (in blue, using PlantCyc databases) and *Raphanus sativus* (in violet, using RadishBase). The numbers in these Venn diagram represent the numbers of shared and unique metabolic pathways and sub-pathways in these species.

Interestingly, these pathways are found to be specific to either only a single Brassica species, or present solely in the Brassica and not in radishes and vice versa (Figure 4 and Table S2). Further studies are needed to explore correlations between such pathways and their corresponding metabolites. Further, while the MEP pathway is present in all four species, there are differences among these species in terms of the enzymes and intermediate reactions of this pathway, which could possibly result in differences in the VOCs. 

### 2.6. Correlations of VOCs

**Figure 5:**
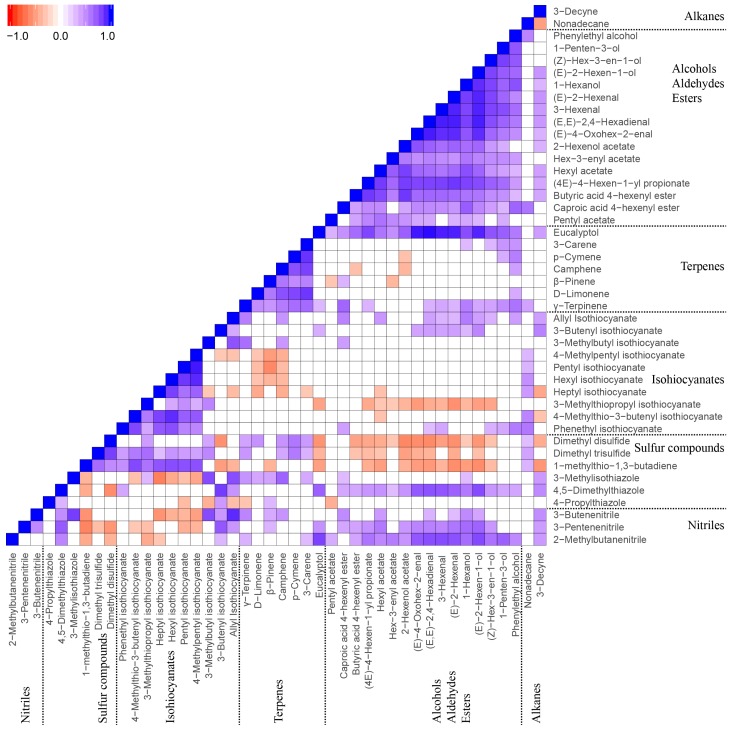
Spearman correlations of identified VOCs in 12 Brassicaceae vegetables. Correlation coefficients (*r*) were in the range of −1 to 1. Only significant correlations (*p* < 0.05) are shown in the heatmap. Blue (*r* = −1) and red (*r* = 1) represent the most significantly negative and positive correlations, respectively. White (0) represents no significant correlation.

The Spearman correlation test was performed to understand the relationships between the identified VOCs. In Figure 5, the significant correlations (*p* < 0.05) of VOCs are presented in blue (positive correlation) and red (negative correlation) respectively, and non-significant correlations are presented in white. The VOCs showed significant positive correlations within their respective chemical groups, which are (a) alcohols/aldehydes/esters (GLVs related VOCs), (b) terpenes, and (c) ITCs. It was noted that the GLVs related VOCs had significant negative correlations with several sulphur compounds (e.g., dimethyl disulphide and dimethyl trisulfide) and showed significant positive correlations with nitriles, but had no significant correlations with most of terpenes and ITCs. Further, terpenes and ITCs did not appear to show any correlation. 

The significant correlations of VOCs with correlation coefficients (r values) larger than 0.8 for positive correlations and lower than −0.5 for negative correlations are highlighted in a network plot (Figure S2). It was obvious that VOCs from the same pathway, such as GLVs from the fatty acid pathway (LOX pathway), terpenes from the MEP pathway, and ITCs from the GLS pathway, had highly positive correlations. In contrast, the VOCs from different pathways either had no correlations or showed negative correlations (e.g., dimethyl disulphide, dimethyl trisulfide, and 3-butenyl isothiocyanate vs. GLVs).

## 3. Discussion

The development and optimization of an untargeted metabolomics method aiming to investigate the volatiles of a biological system represents a crucial step for a successful analysis. HS-SPME followed by GC-HRMS has been shown to be a promising approach in metabolomics studies, and previous studies showed that the SPME fibre with DVB/CAR/PDMS coating phase provided high recovery and reproducibility for analysis of plant VOCs with a wide range of polarity [31]. As plants produce most of the volatiles only after their cells are ruptured, for this study, we first modified a laboratory blender to disintegrate and homogenize the vegetable samples for the production and release of VOCs in an enclosed environment, as shown in Figure S1. After blending, a direct HS-SPME method was employed by exposing a SPME fibre to the headspace of the crushed vegetative tissues in the sealed vessel. There are several advantages of using the present approach. First, the procedure enables capture of the rapid-releasing VOCs soon after the vegetative tissues are being disrupted, reducing VOCs losses during sample pre-treatment in an open environment. Secondly, this approach avoids or minimizes further reactions such as oxidation or reduction of the unstable VOCs. Thirdly, the VOCs captured through the direct HS-SPME method would reflect the metabolic pathways in fresh vegetables more accurately, since it may avoid unexpected breakdown byproducts or metabolites under conditions that disrupt normal metabolic activities of fresh vegetables. In addition, extraction of the VOCs at 37 °C mimics the possible volatile components released and VOCs profiles obtained for the chewing of raw vegetables, such as during the consumption of salads. This newly developed sampling approach represents an alternative simplified means of VOCs sampling from plant tissues.

It has been observed that GLVs can be released from plants within seconds when leaves are injured or suffer from biotic/abiotic stresses, and reach maximum intensity in 30 min [32,33]. Trace amounts of GLVs are usually released by plants under normal physiological conditions, but large amounts can be released under stressed conditions [34]. In recent years, GLVs have been extensively studied and emerged as key players in plant defence as well as plant communication with insects and neighbour plant [35]. For instance, previous studies showed that 3-(Z)-hexenol and its related aldehydes have demonstrated fungicidal and bactericidal activities [36]. In addition to their biological functions, GLVs usually provide fruits and vegetables with their characteristic ‘fresh green’ aroma [35], which are appreciated by consumers and therefore have the potential to be exploited in industrial biotechnology. GLVs are produced by the autolytic oxidative breakdown of membrane lipids including linoleic acid and linolenic acid through the LOX pathway (Figure 3), and their levels varied among plant species and stage of development. In our study, various GLVs were released from green leafy vegetables such as watercress, rocket, kai lan, choy sum, pak choi, broccoli and Brussels sprouts, while much lower levels of these VOCs were emitted from cabbage, Chinese cabbage cauliflower, radish and cherry radish (Figure 2). As the vegetables samples used in this study were mature vegetables, the variations mainly reflected the species-specific differences in VOCs emissions upon vegetative tissue damage. 

Volatile terpenoids originate from the cytosolic mevalonic acid (MVA) pathway (sesquiterpenes, terpenes and geranyl linalool) and the plastidial methylerythritol phosphate (MEP) pathway (monoterpenes) (Figure 3) [1]. Terpenoids play important roles in protecting plants against pests, microbes and pathogens. Monoterpenes and sesquiterpenes are the major volatile compounds released from plants after herbivore damage, attracting arthropods that prey on or parasitize herbivores [8]. Eucalyptol was also found to be a common constituent released from the 12 Brassicaceae vegetables studied. Structurally, eucalyptol is not considered as a terpenoid; however, it has been found to be present at substantial levels during insect-repelling or pesticidal activities [37,38]. Therefore, the results obtained could serve as a basis for the utilization of the volatile compounds from these Brassicaceae vegetables to exploit the specific activities of terpenoid constituents.

In addition to the wide spectrum of volatile compounds mentioned, Brassicaceae vegetables are characterized by GLSs, a unique group of sulphur-containing secondary plant metabolites. When cells are disrupted, myrosinase is released and converts GLSs to various volatile breakdown products including isothiocyanates (ITCs) and nitriles. The breakdown of GLSs and the release of ITCs are of potential significance in several aspects. In Brassicaceae vegetables, ITCs play important roles in the plant’s defence against biotic stressors and act as effective deterrents against a multitude of pathogens due to their antifungal, antimicrobial and insecticidal properties [39]. Moreover, they are important for food sensory properties and have health benefits in the prevention of several human diseases. For example, ITCs are responsible for the sharp taste of mustard and radish [40], and several ITCs have cancer-preventing effects [20]. Among the ITCs, PEITC from watercress has been identified to be a pleiotropic protective agent that interacts with multiple carcinogenic pathways [41,42,43]. 

Many studies have shown the species-specific VOC emissions that reflects different genetic regulations among species [32]. Here we show that the activities of different pathways biosynthesizing volatile metabolites varied among these vegetable species. Evolution of Brassica crops may have been triggered by genome duplication events [44] and syntenic analysis further demonstrated that GLS genes may have increased their copy numbers through whole genome triplication events. In terms of genomic potential for glucosinolate biosynthesis, a pathway that shows major differences in VOCs between these species, there are key differences in the genes detected in these four species. Most of the GLS genes were present in multiple copies and *R. sativus* having 144 GLS genes, which is slightly fewer than the 161 GLS genes present in *Brassica rapa* and, and greater than the 117 GLS genes of *Brassica olereacea* [45]. Although GLS genes are mostly conserved among the Brassicas, some GLS genes differ in the GLS biosynthesis pathways, especially for the short-side-chain aliphatic GLSs. The biosynthesis pathways, of which involve MAM1, MAM3 and CYP79F2 genes that participate in the methionine side-chain elongation step and the AOP2 gene that catalyses the oxidation of aliphatic glucosinolate, present in the radish genome [44]. Finally, the expression levels of MYB28 and MYB29-like genes that are key regulators of glucosinolate biosynthesis in radish could contribute to the different species-specific glucosinolate profiles.

Differences in VOCs associated with glucoerucin, glucoraphanin, glucoraphasatin and glucoraphenin could be due to the presence of GRS1 that is specific to radish. Interestingly, neither AOP2 nor AOP3 was detected in radish, this may lead to the accumulation of glucoraphasatin and glucoraphenin. While epithiospecifier protein (ESP) and nitrile-specifier protein (NSP) can catalyse GSL to produce nitriles and epithionitriles, ESP is inactivated in radish and may lead to, the accumulation of isothiocyanates, such as sulforaphene, instead of epithionitriles or nitriles (Wang et al., 2017), while in *B. rapa* and *B. oleracea*, the major GLS hydrolysis products are nitriles and epithionitriles.

There are 224 and 173 genes in fatty acid and oil biosynthesis in *Brassica rapa* and *Brassica olereacea* respectively, however, the genes in *Raphanus Sativus* for this pathway has not yet been annotated. Most of the metabolites with traumatin and (Z)-3-hexen-1-yl acetate biosynthesis, a sub-pathway in fatty acid biosynthesis and are present in all the species. In brief, the observed differences in VOCs as demonstrated here could shed light on the differences in the biosynthesis pathways for identification of specific enzymes that are activated differentially in these species.

The correlation analysis of various VOCs revealed that the secondary metabolites biosynthesized via the same pathway or originating from a common precursor have close relationship with each other (Figure 5 and Figure S4). Moreover, the three major secondary metabolite pathways (fatty acid-GLVs pathway, MEP-terpenoids pathway, and GLSs-ITCs pathway) appear to operate independently, since only weak or no correlation was observed between the VOCs generated through these three metabolic pathways. This may be due to independent regulation of different metabolic pathways in plants, e.g., by specific transcription factors that control the expression of the genes involved in a specific pathway. It may also reflect different regulation by key enzyme(s) in a pathway, the activity of which controls the availability of metabolite precursors in the pathway or the flow through the downstream pathway. The specific regulation of these pathways could be further investigated to facilitate modern plant breeding and metabolic engineering programs. 

The present study aimed to apply a direct sampling method to characterize VOCs from edible Brassicaceae vegetables. Despite the advantages described above, several limitations exist to this study. First, the effects on VOCs profiles from factors such as growth stage and storage period of these vegetables were not investigated. Secondly, it is noted that the short sample blending time (30 s) could result in incomplete tissue disruption for some vegetables (e.g., cabbage, and Chinese cabbage), which may lead to less reproducible profiles of VOCs. Thirdly, most of the VOCs (7 with standards confirmed) were identified through comparison of mass spectrum with NIST library and further confirmation with authentic standards is needed.

## 4. Materials and Methods

### 4.1. Vegetable Sample Collection and Pre-Treatment

**Figure 6:**
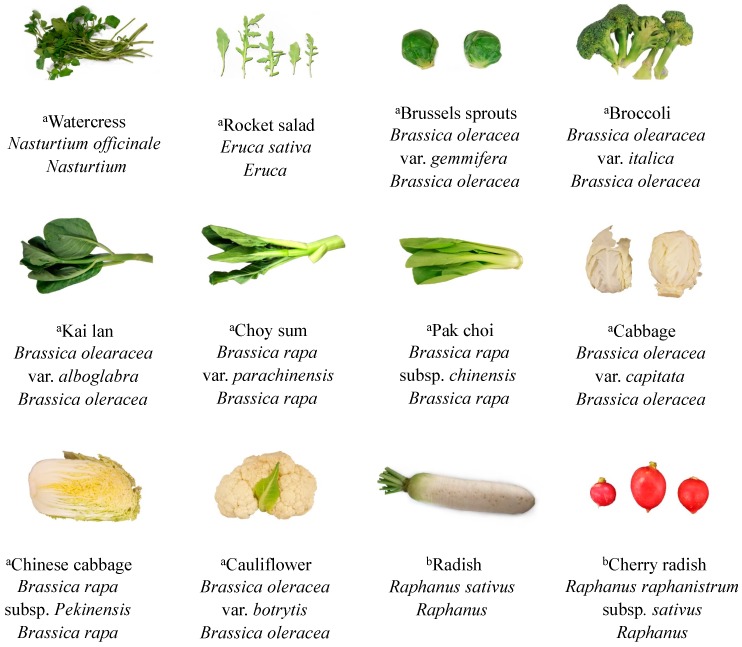
Information (common name, Latin name and species) of the 12 Brassicaceae vegetables (^a^ vegetables grown above-ground; ^b^ vegetables grown below-ground).

Based on earlier studies [16,46] and vegetable consumption patterns in the Asian region, we chose the 12 most commonly consumed Brassicaceae vegetables in this study (Figure 6). These vegetables were purchased from a local supermarket daily and analysed the same day. The edible part of each vegetable was chosen for analysis (leaves: cabbage, Chinese cabbage, Brussels sprouts, pak choi and rocket; leaves and stems: choy sum and kai lan; florets: broccoli and cauliflower; roots: radish and cherry radish.

In this study, a modified laboratory blender (Figure S3) was used to disintegrate and homogenize the vegetable samples. The original cap of the blender was replaced with an air-tight Teflon cap, which can be pierced by a SPME syringe for sample extraction. To simplify the blending process and ensure reproducibility, the vegetables were cut into small pieces prior to blending. The addition of water during blending, extraction temperature and time were optimized for extraction of vegetable VOCs through HS-SPME by using broccoli as a model vegetable. The optimized method was applied to analyse VOCs emitted from the 12 Brassicaceae vegetables. In brief, vegetables were cut into ~2 cm pieces using a stainless scissor (e.g., watercress, rocket, broccoli, kai lan, choy sum, pak choi, Chinese cabbage and cauliflower), and ~2 cm^3^ cubes using a stainless knife (e.g., Brussels sprouts, cabbage, radish and cherry radish), respectively. The vegetable pieces were immediately placed into the blender without addition of water and blended for 30 s with the blender covered with the Teflon cap. After blending, the vessel containing the vegetable sample was put in a water bath (37 °C) through HS-SPME. Four replicates of each vegetable were analysed under optimized conditions. 

### 4.2. HS-SPME and GC-HRMS Analysis

Five authentic standards for metabolite identification, including allyl isothiocyanates, 3-butenyl isothiocyanate, phenethyl isothiocyanate (PEITC), 3-(methylthio) propyl isothiocyanate, and dimethyl disulphide, and the n-alkanes (C8-C20) standard solution for retention index determination, were obtained from Sigma Aldrich (Singapore). 

The procedure of HS-SPME was modified according to the method developed earlier in our lab [47]. The SPME fibre (50/30 DVB/CAR/PDMS, Agilent, USA) was conditioned before use according to the manufacturer’s instruction (270 °C for 1 h). The SPME fibre was exposed into the headspace of the vessel containing the vegetable sample for 30 min. To eliminate potential contamination and capture freshly emitted VOCs, the procedures of SPME fibre pre-condition and sample preparation were optimized to assure that SPME fibre pre-condition was done just before the blending of vegetable sample. After extraction, the SPME fibre was introduced into the GC injector for 30 s at 230 °C in splitless mode for sample injection. 

Agilent 7890A gas chromatography with 7200 quadrupole time-of-flight mass spectrometer (GC-Q-TOF/MS) was used throughout the present study. The separation was performed on DB-5MS column (30 m × 0.25 mm × 0.25 µm, Agilent, USA). Helium was used as the carrier gas. The oven temperature was held at 40 °C for 2 min, and then increased to 185 °C at 5 °C/min and to 300 °C at 30 °C/min, and held for 2 min. The GC total run time was 36.8 min. The starting time of data acquisition was 0.5 min. The ion source was operated in electron ionization (EI) mode at a temperature of 230 °C. The TOF MS was operated in full scan mode and the mass range was from m/z 35 to 500. Mass calibration was conducted after every 5five samples to maintain the high mass accuracy.

It is known that the efficiency of SPME fibre and performance of GC-HRMS may change according to prolong usage. To evaluate the stability of the analytical platform, reference vegetable sample was prepared as QC samples and analysed along with the vegetable samples. To obtain a reference vegetable sample, broccoli, rocket, radish, Chinese cabbage and choy sum (50 g each) were cut into small pieces and blended with 250 mL MilliQ water for 1 min. After centrifugation of the homogenized sample at 3000× *g* for 5 min, 5 mL of the combined supernatant was aliquoted into multiple 20-mL glass vials. The QC samples in the glass vials with magnetic screw caps were stored at −20 °C and routinely analysed through HS-SPME GC-HRMS. The results of the QC samples were evaluated through PCA analysis (Figure S4), which showed that the analytical platform was stable.

### 4.3. Data Analysis and Metabolite Identification

The data analysis protocol was adopted from previous publications with minor changes (Gao et al., 2013; Huang et al., 2013; Zhang et al. 2017). Briefly, the GC-MS data were exported as mzData files in MassHunter Qualitative Analysis software (Agilent, USA) and then uploaded to XCMS online (https://xcmsonline.scripps.edu) for feature extraction and alignment. The aligned features were normalized through the fresh weight of the vegetable samples. After normalization, the aligned features were screened and only the features in any vegetable replicates showing 100% detection frequency (DF) and low relative standard deviation (RSD, <30%) of the peak abundance were kept. The missing values of features (i.e., peak area) were replaced by the half minimum. Principle component analysis (PCA) and orthogonal partial least squares discriminant analysis (OPLS-DA) were performed in SIMCA-P 13.0 with data after logarithmic transformation. A Kruskal‒Wallis test was performed in Multi Experiment Viewer software (MeV). The features with variable importance in projection (VIP) scores above 1.0 in OPLS-DA model and *p* values lower than 0.05 in the Kruskal‒Wallis test were considered to be statistically significant for separating Brassicaceae vegetables. 

The VOCs corresponding to these features were identified through a search in the NIST library. Retention time index was applied to confirm the identification of VOCs. The standards of n-alkanes (C8-C20) dissolved in MilliQ water were analysed following the abovementioned HS-SPME GC-HRMS method. The retention time indices of identified VOCs were calculated and compared with reported values in the NIST library. Spearman correlation analysis for identified VOCs was performed using R version 2.15.2 (2012). 

## 5. Conclusions

In this study, a direct HS-SPME technique combined with non-targeted GC-HRMS metabolomics platform was developed to determine the VOCs profiles in 12 Brassicaceae vegetables. This direct sampling method enables rapid capture of the vegetable VOCs upon tissue rupture. The combination with GC-HRMS analysis yield comprehensive MS spectral information, and have potential applications in plant VOCs analysis. The results demonstrate that edible Brassicaceae could emit a wide spectrum of VOCs for different functions that need to be further determined. The VOCs profiles were found to be able to distinguish the 12 vegetables from the Brassicaceae family. Moreover, correlations among the identified VOCs showed a pathway-independent pattern among the three major VOCs metabolites groups, namely the fatty acid pathway, MEP pathway and glucosinolate pathway. To the best of our knowledge, this is the first investigation using plant VOCs to elucidate the metabolic pathways of a broad range of Brassicaceae vegetables, and the results extend our phytochemical knowledge and provide useful information on chemotaxonomy and VOCs metabolisms of Brassicaceae vegetables.

## Supplementary Materials

The following are available online at http://www.mdpi.com/2218-1989/8/4/94/s1, Figures S1–S4, Table S1.
