# Supplementary material for: Characterization of Plant Volatiles Reveals Distinct Metabolic Profiles and Pathways among 12 Brassicaceae Vegetables
Source: Metabolites. 2018 Dec 14;8(4):94. doi: 10.3390/metabo8040094 (PMC6316591; doi:10.3390/metabo8040094)
Supplement: Supplementary file 1 [file metabolites-08-00094-s001.pdf]

Supplementary Material

Characterization of Plant Volatiles Reveals Distinct Metabolic Profiles and Pathways among 12 Brassicaceae Vegetables

Yu Liu [1], Hui Zhang [1], Shivshankar Umashankar [1], Xu Liang [1], Hui Wen Lee [1], Sanjay Swarup [1] and Choon Nam Ong [1,2,*]

[1] NUS Environment Research Institute, National University of Singapore, Singapore 117411, Singapore; eriliu@nus.edu.sg (Y.L.); erizh@nus.edu.sg (H.Z.); shivshankar.nus@gmail.com (S.U.); eriliaxu@nus.edu.sg (X.L.); erilhw@nus.edu.sg (H.W.L.); sanjay@nus.edu.sg (S.S.)
[2] Saw Swee Hock School of Public Health, National University of Singapore, Singapore 117549, Singapore
* Correspondence: ephocn@nus.edu.sg; Tel.: +65-6516-4982

Table legend:

Table S1. Identified VOCs by GC-Q-TOF/MS.

Figure legends:

Figure S1. Diagram of the sampling device.

Figure S2. Evaluation of QCs reproducibility through PCA analysis.

Figure S3. Representative chromatograms of VOCs emitted from broccoli, (a) comparison of extraction temperature, (b) comparison of extraction time, (c) evaluation of addition of water, and (d) the chromatogram under the optimized conditions.

Figure S4. Network plot of Spearman correlations of VOCs in 12 Brassicaceae vegetables.

Table S1. VOCs identified by GC-Q-TOF/MS.

Compound	RT, min	RI[a]	Mono MW	Formula	CAS	Group	VIP[b]	*p*-Value[c]
3-Decyne	10.47	1001	138.1408	$C_{10}H_{18}$	2384-85-2	Alkane	1.4	5.86×10^{-6}
Nonadecane	22.17	1407	268.313	$C_{19}H_{40}$	629-92-5	Alkane	1.1	8.31×10^{-6}
Phenylethyl Alcohol	14.03	1116	122.0731	$C_8H_{10}O$	60-12-8	Alcohol	1.1	7.83×10^{-6}
1-Penten-3-ol	2.67	<800	86.0732	$C_5H_{10}O$	616-25-1	Alcohol	1.1	2.29×10^{-5}
3-(Z)-Hexenol	6.22	856	100.0188	$C_6H_{12}O$	928-96-1	Alcohol	1.1	2.83×10^{-5}
2-(E)-Hexenol	6.52	867	100.0188	$C_6H_{12}O$	928-95-0	Alcohol	1.0	5.60×10^{-6}
1-Hexanol	6.6	870	100.0188	$C_6H_{12}O$	111-27-3	Alcohol	1.1	6.12×10^{-6}
2-(E)-Hexenal	6.13	852	98.0731	$C_6H_{10}O$	6728-26-3	Aldehyde	1.2	7.86×10^{-6}
3-Hexenal	4.72	<800	98.0731	$C_6H_{10}O$	4440-65-7	Aldehyde	1.2	8.69×10^{-6}
2,4-(E,E)-Hexadienal	7.82	914	96.0575	C_6H_8O	142-83-6	Aldehyde	1.3	3.84×10^{-6}
(E)-4-Oxohex-2-enal	9.39	965	112.0524	$C_6H_8O_2$	2492-43-5	Aldehyde	1.3	7.79×10^{-6}
2-Hexenol acetate	11.05	1019	142.0994	$C_8H_{14}O_2$	10094-40-3	Ester	1.24	1.75×10^{-5}
(3Z)-3-Hexenyl acetate	10.75	1010	142.0993	$C_8H_{14}O_2$	1708-82-3	Ester	1.6	1.35×10^{-4}
Hexyl acetate	10.98	1017	144.1150	$C_8H_{16}O_2$	142-92-7	Ester	1.3	8.38×10^{-6}
(4E)-4-Hexenyl propionate	13.6	1102	156.1150	$C_9H_{16}O_2$	-	Ester	1.2	3.57×10^{-6}
(4E)-4-Hexenyl butyrate	14.93	1147	170.1307	$C_{10}H_{18}O_2$	69727-41-9	Ester	1.3	1.45×10^{-5}
(4E)-4-Hexenyl hexanoate	20.58	1347	198.1620	$C_{12}H_{22}O_2$	88552-98-1	Ester	1.3	6.28×10^{-6}
Pentyl acetate	7.9	917	130.0993	$C_7H_{14}O_2$	628-63-7	Ester	1.3	2.14×10^{-5}
Eucalyptol	11.54	1035	154.1358	$C_{10}H_{18}O$	470-82-6	Monoterpene	1.2	7.08×10^{-6}
3-Carene	8.47	936	136.1252	$C_{10}H_{16}$	13466-78-9	Monoterpene	1.0	1.38×10^{-5}
p-Cymene	11.32	1028	134.1095	$C_{10}H_{14}$	99-87-6	Monoterpene	1.4	1.26×10^{-5}
Camphene	8.97	1052	136.1252	$C_{10}H_{16}$	79-92-5	Monoterpene	1.2	6.12×10^{-6}
β-Pinene	10.24	993	136.1252	$C_{10}H_{16}$	127-91-3	Monoterpene	1.3	7.83×10^{-6}
D-Limonene	11.46	1033	136.1252	$C_{10}H_{16}$	5989-27-5	Monoterpene	1.6	5.94×10^{-6}
γ-Terpinene	12.36	1061	136.1252	$C_{10}H_{16}$	99-85-4	Monoterpene	1.2	1.12×10^{-5}
Allyl Isothiocyanate	6.9	882	99.0142	C_4H_5NS	57-06-7	Isothiocyanate	1.7	3.82×10^{-6}

3-Butenyl isothiocyanate	9.905	982	113.0299	C_5H_7NS	3386-97-8	Isothiocyanate	1.7	4.38×10^{-6}
3-Methylbutyl isothiocyanate	12.29	960	129.0612	$C_6H_{11}NS$	628-03-5	Isothiocyanate	1.4	4.69×10^{-6}
4-Methylpentyl isothiocyanate	15.41	1163	143.0769	$C_7H_{13}NS$	17608-07-0	Isothiocyanate	1.1	5.98×10^{-6}
Pentyl isothiocyanate	13.47	1098	129.0612	$C_6H_{11}NS$	629-12-9	Isothiocyanate	1.3	6.20×10^{-6}
Hexyl isothiocyanate	16.51	1200	143.0769	$C_7H_{13}NS$	4404-45-9	Isothiocyanate	1.1	3.16×10^{-6}
Heptyl isothiocyanate	18.33	1264	157.0925	$C_8H_{15}NS$	4426-83-9	Isothiocyanate	1.2	4.22×10^{-6}
3-Methylthiopropyl isothiocyanate	19.64	1311	147.0176	$C_5H_9NS_2$	505-79-3	Isothiocyanate	1.5	7.83×10^{-6}
4-Methylthio-3-butenyl isothiocyanate	22.46	1419	159.0176	$C_6H_9NS_2$	51598-96-0	Isothiocyanate	1.2	6.34×10^{-6}
Phenethyl isothiocyanate	23.7	1467	163.0456	C_9H_9NS	2257-09-2	Isothiocyanate	1.3	5.53×10^{-6}
Dimethyl disulfide	3.58	<800	93.9911	$C_2H_6S_2$	624-92-0	Sulphur compounds	1.1	5.39×10^{-6}
Dimethyl trisulfide	9.51	969	125.9631	$C_2H_6S_3$	3658-80-8	Sulphur compounds	1.0	7.60×10^{-6}
1-(Methylsulfanyl)-1,3-butadiene	5.86	842	100.0347	C_5H_8S	10574-97-7	Sulphur compounds	1.0	2.26×10^{-6}
3-Methylisothiazole	10.64	1006	99.0143	C_4H_5NS	693-92-5	Sulphur compounds	1.5	9.45×10^{-6}
4,5-Dimethylthiazole	14.245	1124	113.0299	C_5H_7NS	3581-91-7	Sulphur compounds	1.6	4.12×10^{-6}
4-Propylthiazole	13.01	1083	127.0456	C_6H_9NS	41981-60-6	Sulphur compounds	1.6	3.30×10^{-5}
3-Butenenitrile	2.38	<800	67.0422	C_4H_5N	109-75-1	Nitrile	1.5	5.21×10^{-6}
3-Pentenenitrile	3.955	<800	81.0578	C_5H_7N	4635-87-4	Nitrile	1.4	1.01×10^{-5}
2-Methylbutanenitrile	5.81	840	83.0735	C_5H_9N	18936-17-9	Nitrile	1.2	1.51×10^{-4}

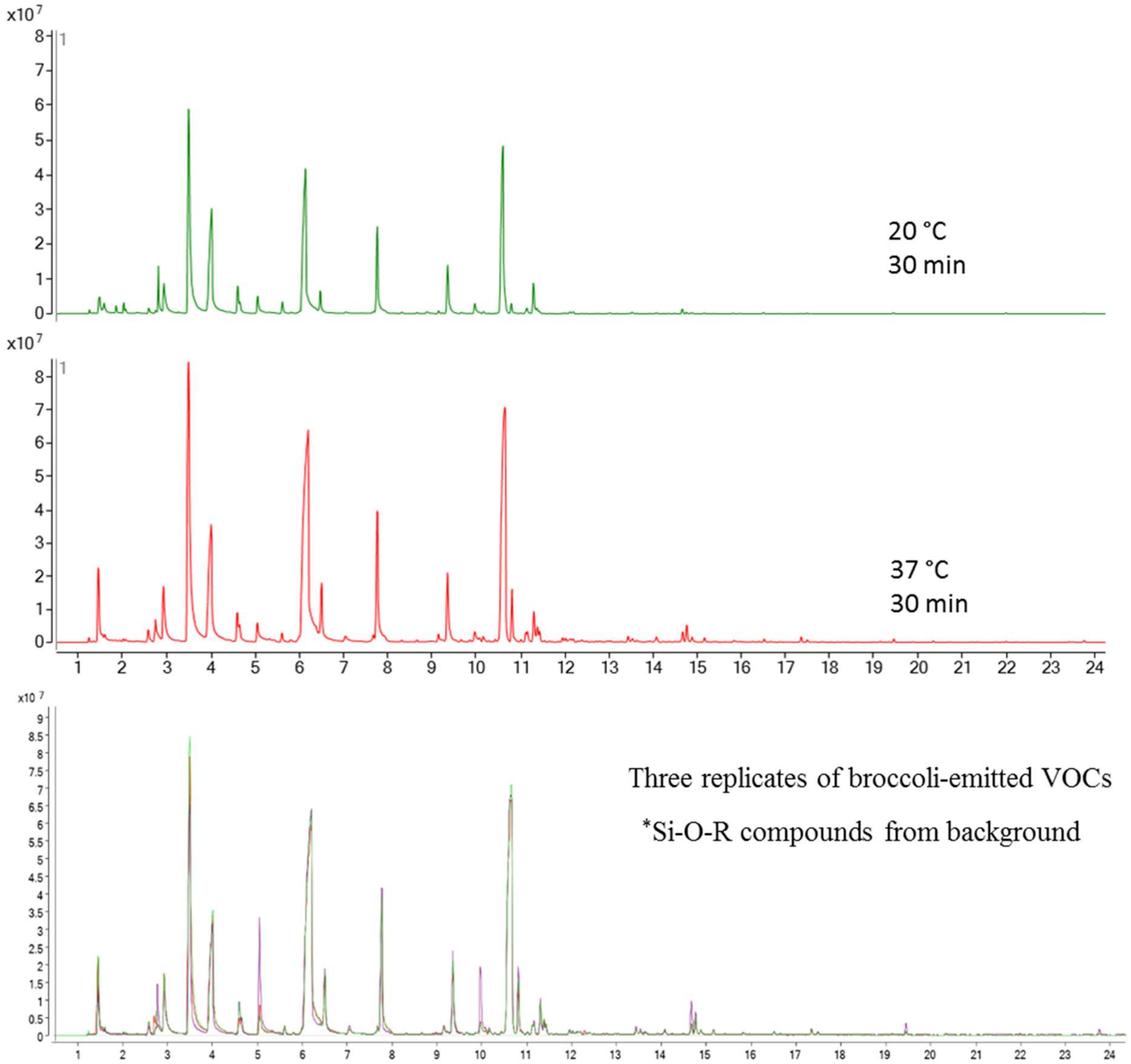

Figure S1a. Comparison of extraction temperature.

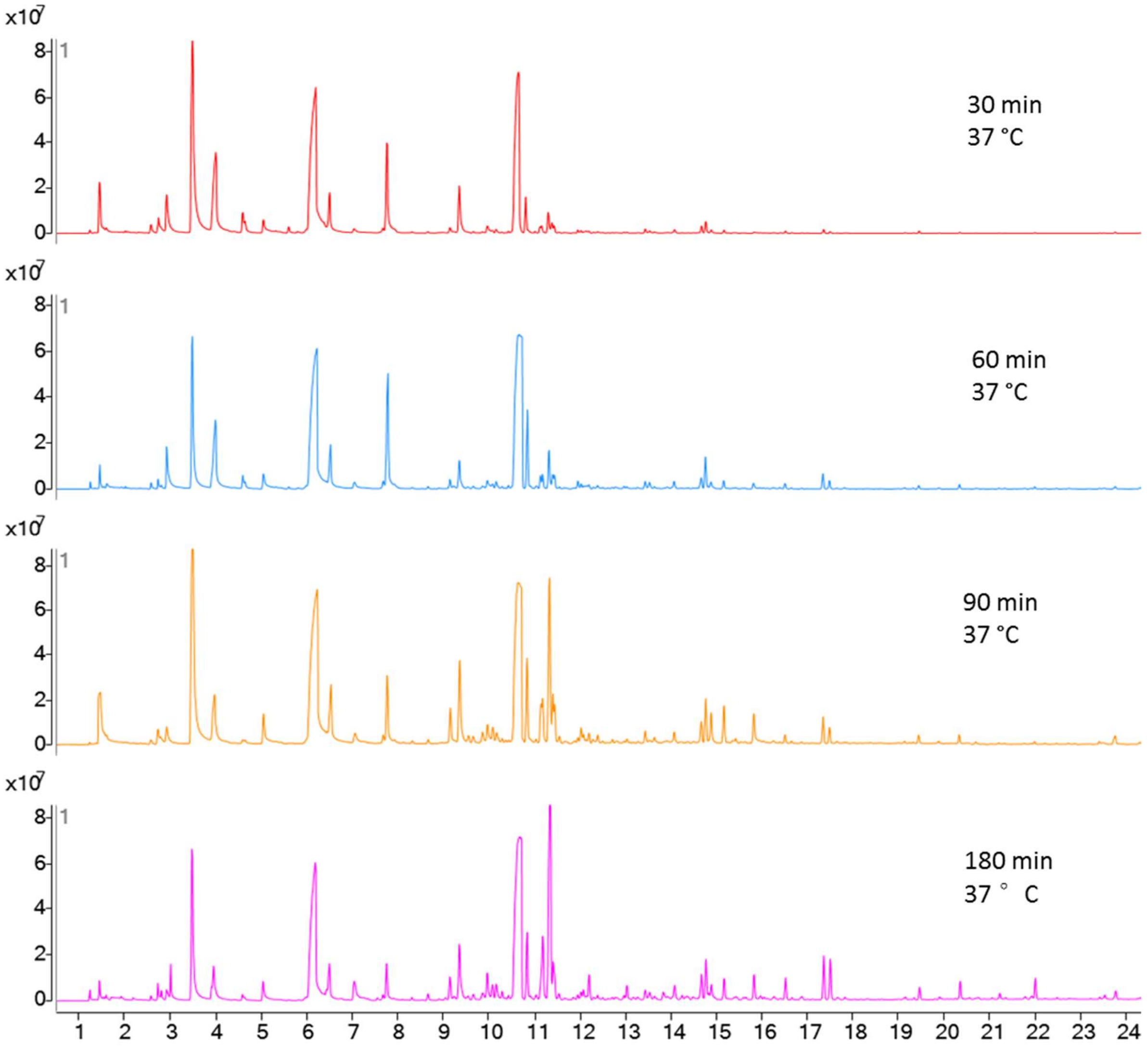

22

Figure S1b. Comparison of extraction time.

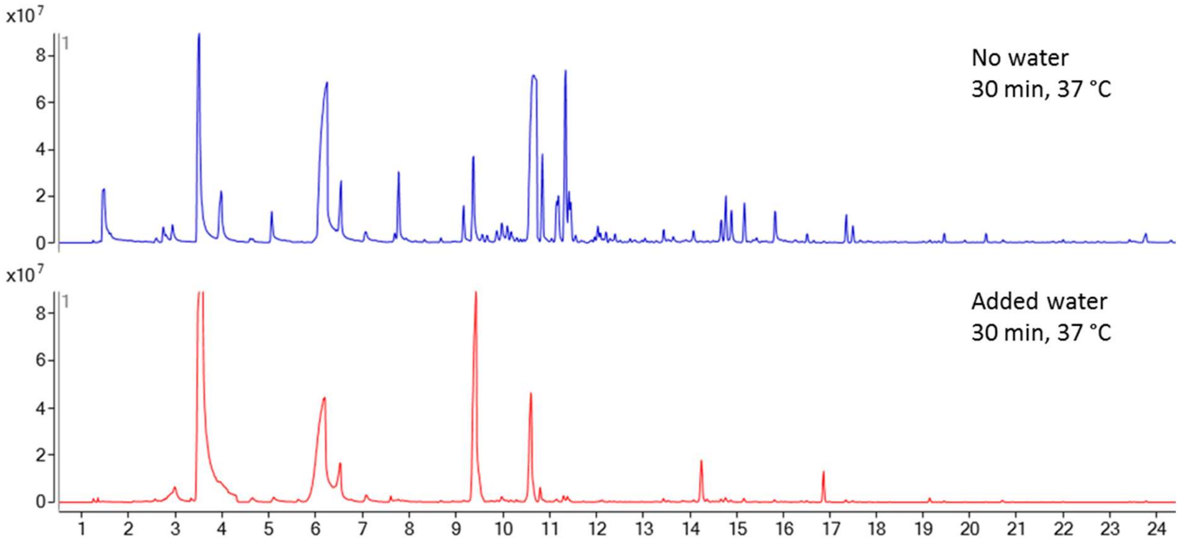

Figure S1c. Evaluation of addition of water.

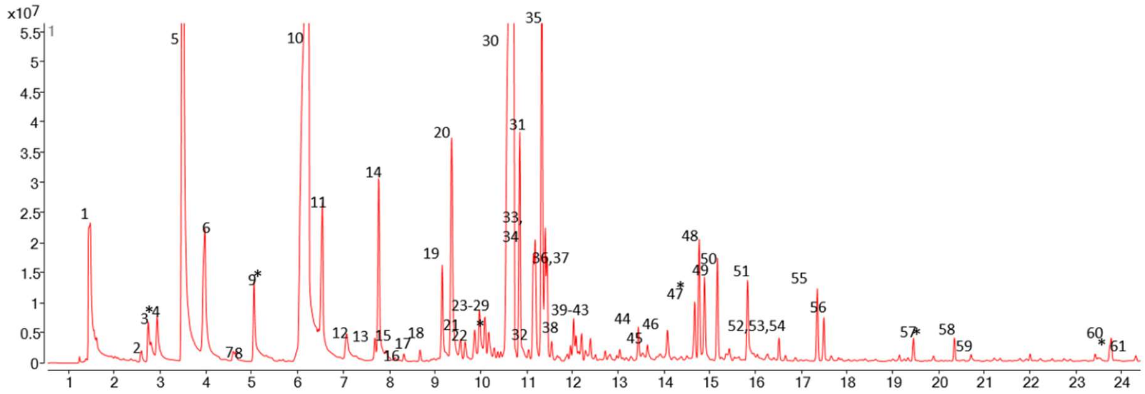

1. Acetone,
2. 1-Penten-3-ol,
3. Si-O-R from SPME fiber
4. Methyl isothiocyanate,
5. Disulfide, dimethyl,
6. 1-Pentanol
7. 3-Hexenal,
8. 1.2-Methylcyclopentanol,
9. Si-O-R from SPME fiber
10. 3-Hexen-1-ol, (E)-,
11. 1-Hexanol,
12. p-Xylene,
13. 2-Penten-1-ol, acetate, (Z)-,
14. Acetic acid, pentyl ester,
15.4-Penten-1-ol, 3-methyl-, acetate,
17. 3-Carene,
18. 3-Decyne,
19. Octane, 2,2,6-trimethyl-,
20. Dimethyl trisulfide,
21. Pentane, 2,2,3,4-tetramethyl-,
22. Tetradecane, 1-chloro-,
23. Heptane, 2,2,3,5-tetramethyl-,
24. Si-O-R from SPME fiber
25. Decane, 2,6,7-trimethyl-,
26. Benzene, 1-ethyl-3-methyl-,
27. Bicyclo[3.1.1]heptane, 2,6,6-trimethyl-, [1R-(1α,2α,5α)]-,
28. Octane, 2,6,6-trimethyl-,
29. Tetradecane,
30. 4-Hexen-1-ol, (4E)-, acetate,
31. Acetic acid, hexyl ester,
32. Benzene, 1,2,3-trimethyl-,
33.Heptane, 4-ethyl-2,2,6,6-tetramethyl-,
34. β-Cymene,
35. D-Limonene,
36. Eucalyptol,
37. Hexane, 3,3-dimethyl-,
38. Undecane, 3,6-dimethyl-,
39. Propanoic acid, 2-methyl-, pentyl ester,
40. Heptane, 5-ethyl-2,2,3-trimethyl-,
41. γ-Terpinene,
42. 1,2,6-Hexanetriol,
43. Undecane, 3-methyl-,
44. Propanoic acid, 4-hexen-1-yl ester, 45. 3-Nonen-1-ol, (Z)-,
46. Isophorone,
47. Si-O-R from SPME fiber
48. Butanoic acid,4-hexen-1-yl ester,
49. Camphor,
50. l-Menthone,
51. Levomenthol,
52. Cyclopentane, 1-hexyl-3-methyl-,
53. Nonadecane,
54. 8-Dodecenol,
55. Pentanoic acid, 4-hexen-1-yl ester, 56. n-Valeric acid cis-3-hexenyl ester,
57. Si-O-R from SPME fiber
58. Dodecanoic acid, 2-hexen-1-yl ester,
59. 5-Keto-2,2-dimethylheptanoic acid, ethyl(ester),
60. Si-O-R from SPME fiber
61. Guaia-1(10),11-diene,

Figure S1d. The chromatogram under the optimized conditions.

Figure S1. Representative chromatograms of VOCs emitted from broccoli: (**a**) comparison of extraction temperature, (**b**) comparison of extraction time, (**c**) evaluation of addition of water, and (**d**) the chromatogram under the optimized conditions.

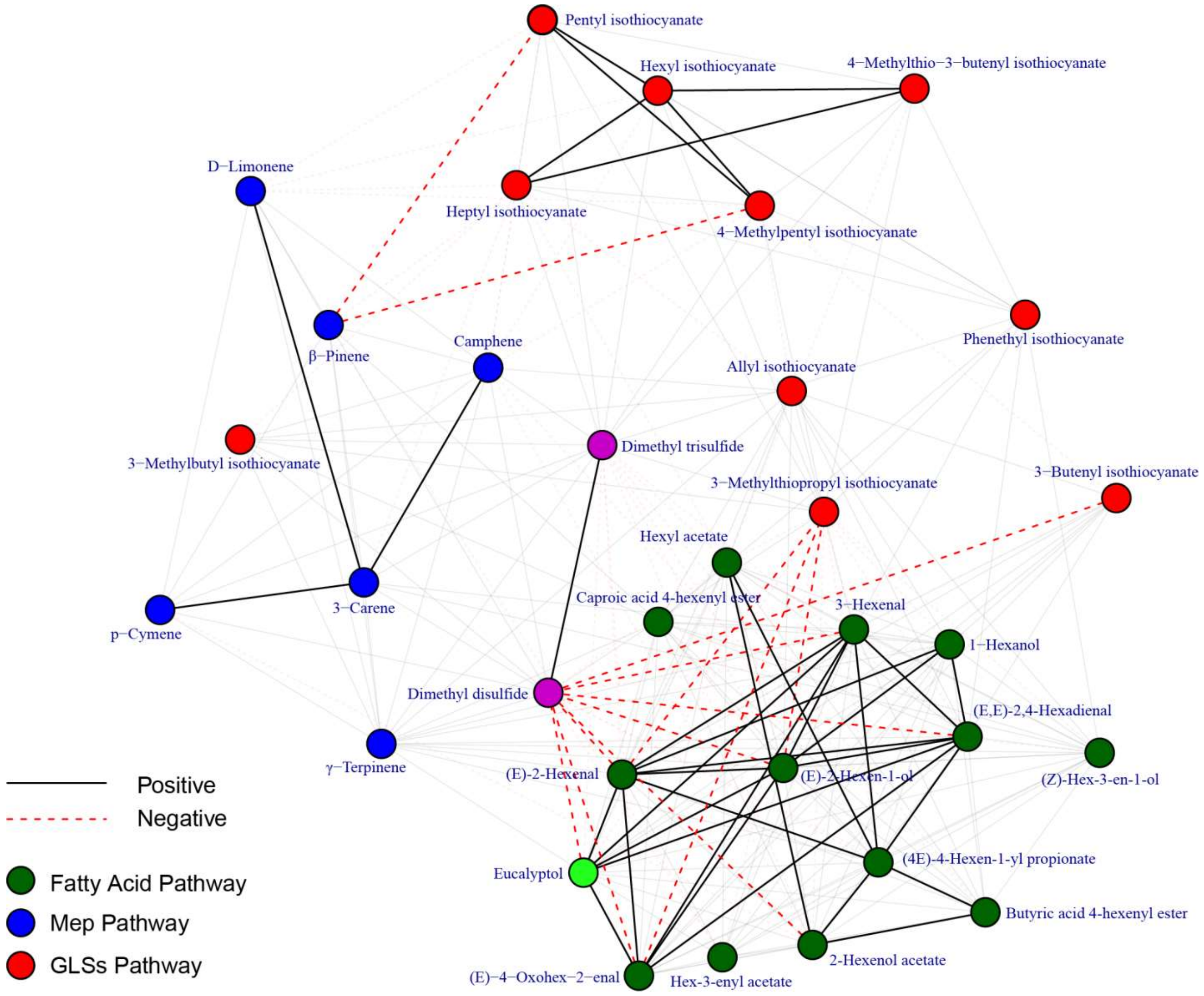

Note: Significant correlations ($p < 0.05$) were plotted between VOCs. The positive correlation with a correlation coefficient higher than 0.8 is shown by the black solid line. The negative correlation with a correlation coefficient lower than –0.5 is shown by the red dashed line.

Figure S2. Network plot of Spearman correlations of VOCs in 12 Brassicaceae vegetables.

26

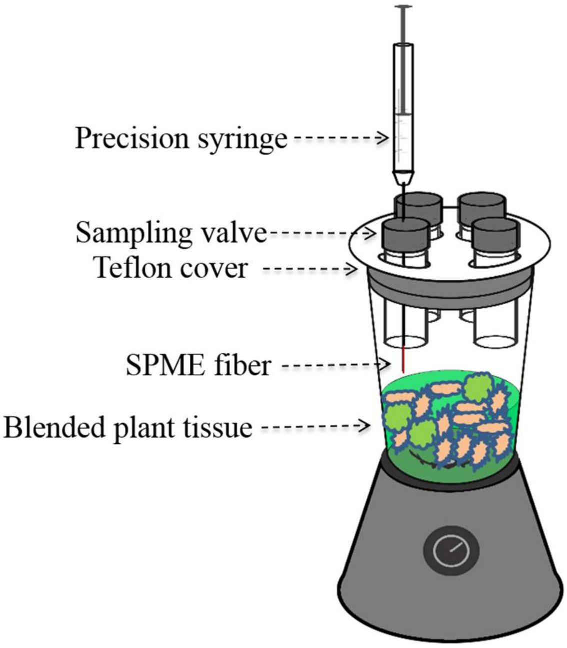

Figure S3. Diagram of the sampling device.

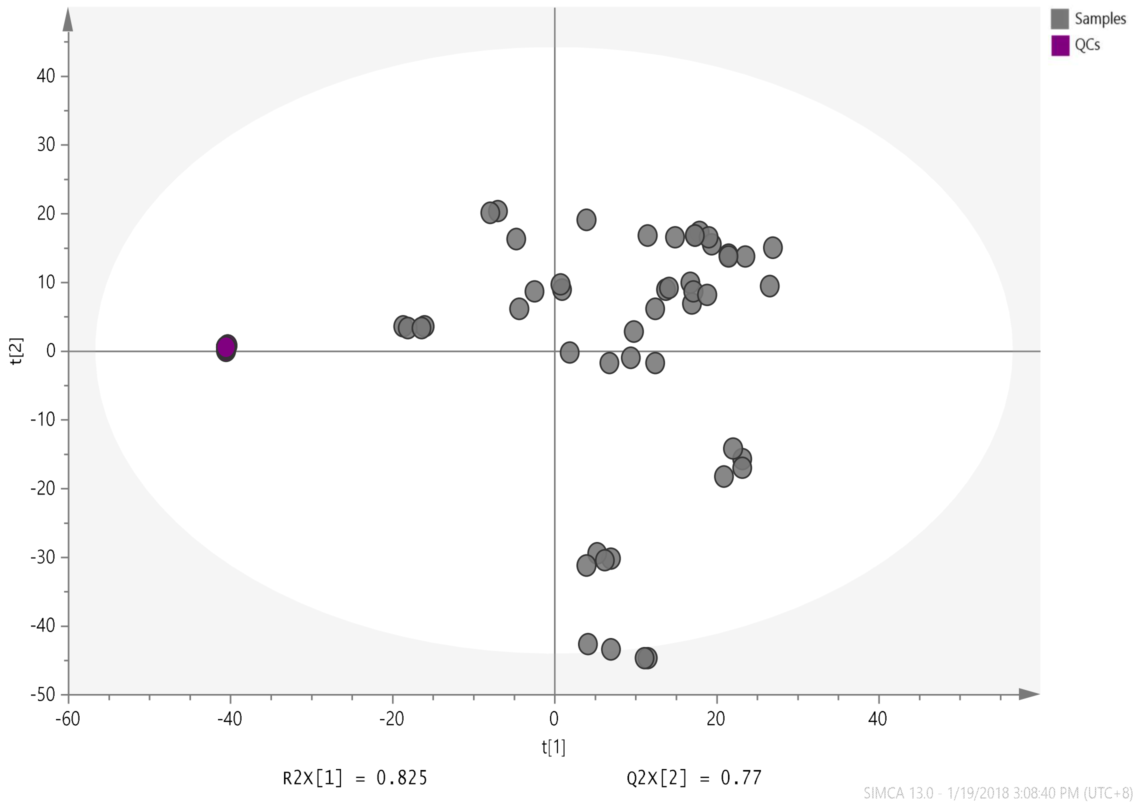

Figure S4. Evaluation of QCs' reproducibility through PCA analysis.
